# Principal Component Analysis Based Quaternion-Valued Medians for Non-Invasive Blood Glucose Estimation

**DOI:** 10.3390/s25123746

**Published:** 2025-06-15

**Authors:** Jingheng Feng, Bingo Wing-Kuen Ling

**Affiliations:** School of Information Engineering, Guangdong University of Technology, Guangzhou 510006, China; 2112203039@mail2.gdut.edu.cn

**Keywords:** quaternion-valued median, principal components analysis, non-invasive blood glucose estimation

## Abstract

For four-channel photoplethysmograms (PPGs), this paper employs quaternion-valued medians as features for performing non-invasive blood glucose estimation. However, as the PPGs are contaminated by noise, the quaternion-valued medians are also contaminated by noise. To address this issue, principal component analysis (PCA) is employed for performing the denoising. In particular, the covariance matrix of the four-channel PPGs is computed and the eigen vectors of the covariance matrix are found. Then, the quaternion-valued medians of the four-channel PPGs are found and these quaternion-valued medians are represented as the four-channel real-valued vectors. By applying the PCA to these four-channel real-valued vectors and reconstructing the denoised four-dimensional real-valued vectors, these four-dimensional real-valued vectors are denoised. Next, these denoised four-dimensional real-valued vectors are represented as the denoised quaternion-valued medians. Compared to the traditional denoising methods and the traditional feature extraction methods that are performed in the individual channels, the quaternion-valued medians and the PCA are computed via fusing all of these four-channel PPGs together. Hence, the hidden relationships among these four channels of the PPGs are exploited. Finally, the random forest is used to estimate the blood glucose levels (BGLs). Our proposed PCA-based quaternion-valued medians are compared to the median of each channel of the PPGs and other features such as the time-domain features and the frequency-domain features. Here, the effectiveness and robustness of our proposed method is demonstrated using two datasets. The computer numerical simulation results indicate that our proposed PCA-based quaternion-valued medians outperform the existing quaternion-valued medians and the other features for performing non-invasive blood glucose estimation.

## 1. Introduction

Diabetes impacts over 10% of the global population [1]. It exists in various types. Although each type of diabetes has its distinct primary causes, they all lead to an increase in BGLs and associated complications. These complications can result in serious health issues [2]. In fact, the chance of having complications can be significantly reduced if the BGLs are monitored continuously and controlled in time. Therefore, monitoring BGLs continuously plays an important role in the diagnosis of the diabetes.

However, diabetes is a chronic disease. It requires continuous monitoring of BGLs. This introduces high pressure on the national medical system and the national transportation system, and reduces the national productivity if the continuous blood glucose estimation is performed in clinics or hospitals. To address this issue, continuous blood glucose estimation needs to be performed at home or at the workplace. The common continuous blood glucose estimation method is via the invasive approach. Nevertheless, the invasive blood glucose estimation approach causes pain to the subjects and has a risk of infection.

In order to address these drawbacks, various non-invasive blood glucose estimation methods have been developed. In particular, the PPGs are acquired from the fingertip based on the near infrared (NIR) spectroscopy approach [3]. Then, the features are extracted from the PPGs and the BGLs are estimated using the machine learning models. Here, many methods are developed for extracting the features from the single-channel PPGs. They include statistical features such as the skewness and the energy of the PPGs [4]. In addition, the logarithmic energy entropy, the Kaiser Teager energy (KTE), the spectral entropy, and the auto-regressive (AR) coefficients [5,6] are also employed as the features. By integrating the various types of features together, the physiological characteristics of the PPGs such as the shape, the spectral characteristics, and the energy distribution of the envelope of the PPGs can be effectively described. Hence, the relationship between the PPGs and the BGLs can be more comprehensively exploited. As a result, these features are extensively employed in the estimation of BGLs [7]. Moreover, the features extracted from the mel frequency cepstral coefficients (MFCCs) are also employed as the features. These features provide additional insight for understanding the small variations in the time frequency characteristics of the PPGs. This is crucial for accurately detecting and monitoring the changes in the BGLs [8]. Furthermore, the features extracted from the heart rate variability (HRV) are also utilized to evaluate the degree of the sympathetic tone. This provides comprehensive physiological information for monitoring the BGLs [9]. Since these approaches are easy to implement, several wearable blood glucose monitoring devices have been developed in recent years using these approaches, see [6,10,11]. It is worth noting that the impedance spectroscopy approach can achieve 56% of all of the estimated values in region A of the Clarke error grid [10]. Similarly, the impedance and the multi-wavelength NIR spectroscopy approach can achieve the mean absolute relative difference (MARD) at 20% and the proportion of all of the estimated values in region A of the Clarke error grid at 60% [11]. However, these methods are based on the single-channel PPGs. Hence, the achieved accuracy is limited and it often fails to meet the accuracy and the reliability requirements in many practical situations.

To address the above issues, multi-channel PPG acquisition devices have been developed [4,7,9]. The existing methods for extracting features from multi-channel PPG signals typically process each channel independently, extracting features from individual channels separately before merging them into a single feature vector. For example, Tsai et al. collected dual-channel PPG signals from the finger and wrist, extracting time-domain and frequency-domain features independently from each channel [4]. Similarly, Gupta et al. obtained red, green, and infrared wavelength PPG signals from the finger and extracted independent features from each wavelength channel [7]. Wei et al. collected two PPG signals of the same wavelength from the finger and extracted heart rate variability (HRV) features independently for each [9]. While these approaches benefit from the richer information provided by multi-channel signals compared to single-channel acquisition and are relatively simple and intuitive, they exhibit notable limitations. Specifically, because the feature extraction is performed on a per-channel basis, these methods cannot effectively exploit the coupling or interdependence among different PPG channels [12]. This inter-channel information may carry important physiological insights that are otherwise lost in independent processing pipelines.

It is worth noting that quaternion-valued classification techniques have been increasingly employed for processing multi-channel human physiological signals to improve predictive performance [13,14]. Compared with traditional real-valued or complex-valued approaches, quaternion-valued representations offer a unique advantage in modeling multidimensional inter-channel correlations within a compact and unified mathematical framework. This capability is particularly beneficial for multi-channel signal fusion and joint feature extraction. For example, in [13], EEG signals were first decomposed into four canonical brain wave components (delta, theta, alpha, and beta) using fast Fourier transform (FFT), and then modeled as quaternion-valued signals to preserve the intrinsic cross-band dependencies. Subsequently, quaternion-valued singular spectrum analysis (QSSA) was applied for feature extraction, followed by classification for sleep staging. This method achieved a classification accuracy of 97.5%, which outperformed several existing real-valued approaches, demonstrating the effectiveness of quaternion-based modeling in capturing both spatial and spectral features. In another example [14], nine optical path-length NIR spectral signals were acquired using near-infrared spectroscopy, and three channels were randomly selected and encoded as a quaternion-valued signal to preserve inter-channel spectral relationships. Quaternion principal component analysis (QPCA) was then employed to extract features jointly from the three channels, followed by regression using support vector regression (SVR). This approach significantly outperformed traditional single-wavelength PCA-based models, achieving a correlation coefficient of 0.9854. These two applications demonstrate the effectiveness and flexibility of quaternion-valued signal processing techniques in handling multi-channel signals, particularly in scenarios where preserving inter-channel dependencies is essential for accurate analysis.

This paper proposes a non-invasive approach for performing blood glucose monitoring via a wearable device and a smartphone. Hence, it can provide a domestic solution for performing continuous blood glucose estimation and diabetes management. The wearable device consists of various PPG sensors. Since the consumable is not required, this non-invasive approach is more cost effective in the long term compared to the invasive blood glucose monitoring devices. In addition, our proposed method has a low computational complexity and can be implemented on consumer-grade hardware such as smartphones and wearable devices.

The major contributions and the novelties of this paper are as follows. First, this paper employs the quaternion-valued medians as the features for performing non-invasive blood glucose estimation. Second, this paper proposes performing the denoising via applying the PCA to the quaternion-valued medians instead of performing the denoising on the four-channel PPGs. The computer numerical simulation results demonstrate that our proposed method yields higher regression accuracy compared to the existing methods for performing non-invasive blood glucose estimation. This demonstrates the effectiveness and robustness of our proposed method. The outline of this paper is as follows. Section 2 briefly reviews the PCA and the quaternion-valued theory. Section 3 presents our proposed method. Section 4 presents the computer numerical simulation results. Finally, Section 5 draws a conclusion.

## 2. Reviews on the PCA and the Quaternion-Valued Theory

### 2.1. PCA

PCA is the most common linear method for performing dimension reduction and feature denoising [15]. In particular, PCA generates a new set of orthogonal vectors known as principal components. Then, the raw data are projected to these principal components. Here, only the projected components with large variances are retained, while the projected components with small variances are discarded. In this case, the key information from the raw data is retained, but the noise in the data is suppressed. Since the dimensions of the projected vectors are reduced [16], a set of the low-dimensional vectors is obtained.

The basic operations of the PCA are as follows. Let n be the total number of the feature vectors. Let X(j) be the *j*th feature vector. First, the decentralization is performed. That is,

(1) $$\bar{X}(i)=X(i)-\frac{1}{n}\sum_{j=1}^{n}X(j)$$

Let $\bar{X}$ be the decentralized data matrix. That is, X(j) is the *j*th column of $\bar{X}$. Let Ω be the covariance matrix of $\bar{X}$. By performing the singular value decomposition on Ω, let U be the unitary matrix with its columns being the singular vectors and Λ be the diagonal matrix with its diagonal elements being the singular values. That is,

(2) $$\Omega =\frac{1}{n}\bar{X}{\bar{X}}^{T}=U\Lambda U^{T}$$

Let m be the dimensions of the truncated projected feature vectors. Let $u_{p}$ be the *p*th singular vector. Let $U_{A}=\left[\begin{array}{ccc} u_{1} & \cdots & u_{m} \end{array}\right]$ be the matrix containing the first m columns of U. Let $\theta =diag\left(\lambda_{1},\lambda_{2},\cdots ,\lambda_{m}\right)$ be the diagonal matrix containing the first m eigenvalues. Let Z be the matrix with its columns being the truncated projected feature vectors. That is,

(3) $$Z=U_{A}^{T}\bar{X}$$

Then, the covariance matrix of Z is θ.

The fundamental assumption of PCA is that the original data matrix is full rank. Hence, the covariance matrix of the original feature vectors is strictly positive definite. In this case, the eigen vectors of the covariance matrix are orthogonal to one another. Therefore, these eigen vectors form a set of the principal components. Here, each principal component represents the primary coordinate axis of the new coordinate system with the variances of the original feature vectors projected to these principal components being sorted in the descending order. By projecting the original high-dimensional feature vectors to some of these principal components, the original feature vectors in the high-dimension space are transformed to the new feature vectors in the low-dimensional space. Hence, the dimension of the original feature vectors is reduced. Also, as the projection operation is linear, the projected feature vectors are the linear combination of the principal components. It is worth noting that these principal components can be categorized into two types. One includes the information-dominant principal components and another includes the noise-dominant principal components. Since the principal components corresponding to low variances are ignored, the noise-dominant principal components are removed. Hence, PCA can suppress the noise in the feature vectors. In general, if the signal-to-noise ratios (SNRs) of the principal components are larger than 0 dB, then these principal components should be retained. Otherwise, they should be discarded. However, it is difficult to determine the SNRs of the individual principal components. Hence, selecting the principal components is critical for the PCA-based denoising algorithm. This paper aims to address this issue by conducting comparative simulations.

### 2.2. Quaternion-Valued Theory

#### 2.2.1. Quaternion-Valued Algebra

Let ℍ be the set of the quaternion-valued numbers and q be a quaternion-valued number. That is, q∈ℍ. Let $ℜ\left(q\right)$ and $ℑ\left(q\right)$ be the real part and the imaginary part of q, respectively. Since the quaternion-valued numbers have three imaginary components, let r, x, y, and z be the real component, the i component, the j component, and the k component of q, respectively. That is,

(4) $$q=ℜ\left(q\right)+ℑ\left(q\right)=r+xi+yj+zk$$

where $ℜ\left(q\right)=r$ and $ℑ\left(q\right)=xi+yj+zk$.

The algebraic operations of the quaternion-valued numbers obey the following rules:

(5a) ij=k

(5b) jk=i

(5c) ki=j

(5d) ji=−k

(5e) kj=−i

(5f) ik=−j

and

(5g) $$ijk=i^{2}=j^{2}=-1$$

Let $q_{1}$ and $q_{2}$ be two quaternion-valued numbers. The distance between $q_{1}$ and $q_{2}$ is defined as the minimum between $∥q_{1}-q_{2}∥$ and $∥q_{1}+q_{2}∥$ [17]. That is,

(6) $$d=\min(∥q_{1}-q_{2}∥,∥q_{1}+q_{2}∥)$$

#### 2.2.2. Quaternion-Valued Medians

For a one-dimensional time series, the typical method of finding its median is to sort its values in the ascending order and take the middle value in the sorted sequence as the median. Let m(·) be the above median operator. However, when each component in the quaternion-valued sequence is sorted in the ascending order, the locations of the medians of the individual components of the quaternion-valued sequence are not necessarily the same. Hence, this approach is not applied to find the median of a quaternion-valued sequence.

Let q be a quaternion-valued sequence. Let $q_{r}$, $q_{x}$, $q_{y}$, and $q_{z}$ be the real component, the i component, the j component, and the k component of q, respectively. Let $X_{M}$ be the quaternion-valued number such that

(7) $$X_{M}=m(q_{r})1+m(q_{x})i+m(q_{y})j+m(q_{z})k$$

Obviously, $X_{M}$ can be used as a quaternion-valued median.

Moreover, let d(⋅,⋅) be a distance function between two quaternion-valued numbers. Let n be the total number of the points in a quaternion-valued sequence. Let $q_{1},\cdots ,q_{n}$ be this quaternion-valued sequence. To determine the median of this quaternion-valued sequence, a commonly used approach involves selecting a quaternion-valued number from this sequence such that the cumulative distance between it and all other quaternion-valued numbers in this sequence is minimized [18]. Let

(8) $$X_{O}=\underset{q\in \{q_{1},q_{2},\ldots ,q_{n}\}}{\min}\sum_{i=1}^{n}d(q,q_{i})$$

be this quaternion-valued number. Obviously, it can be used as a quaternion-valued median of this sequence. In fact, this approach is widely used for finding a median pixel in an image with four color planes.

Furthermore, since the quaternion-valued numbers can be used to describe the rotations of the objects, the quaternion-valued numbers are normalized to the unit quaternion-valued numbers before finding the median of the quaternion-valued sequence. Let

(9) $$X_{K}=\underset{q\in \{q_{1},q_{2},\ldots ,q_{n}\}}{\min}\sum_{i=1}^{n}d(\frac{q}{\left\|q\right\|},\frac{q_{i}}{\left\|q_{i}\right\|})$$

be this quaternion-valued number. Obviously, it can also be used as a quaternion-valued median of the quaternion-valued sequence.

In addition, the geometric median is a quaternion-valued number that minimizes the cumulative distance between it and all other quaternion-valued numbers in this sequence. Let

(10) $$X_{G}=\underset{q\in ℍ}{\min}\sum_{i=1}^{n}d(q,q_{i})$$

be this quaternion-valued number. Obviously, it represents the central location of the quaternion-valued sequence. Hence, it can also be used as a quaternion-valued median of the quaternion-valued sequence. To find the solution of this minimization problem, various algorithms have been proposed [19].

## 3. Our Proposed Method

**Figure 1:**
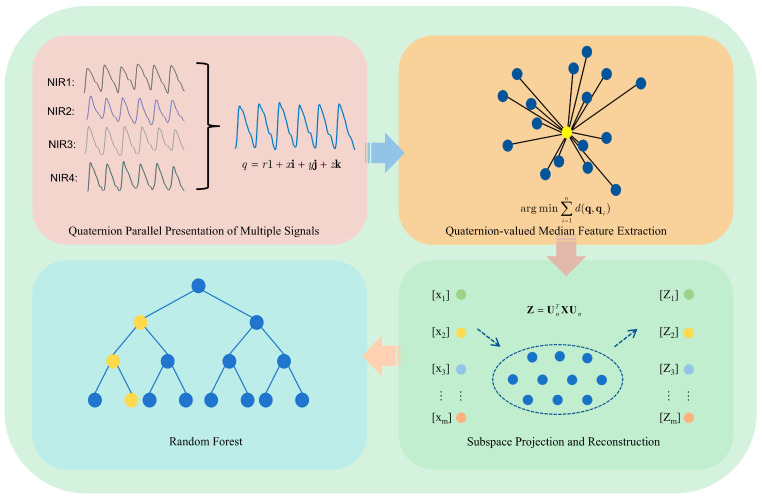
The framework of our proposed algorithm.

Figure 1 illustrates the framework of our proposed algorithm. First, four NIR sensors are employed for acquiring the four-channel PPGs. Second, the quaternion-valued PPGs are formed and the existing quaternion-valued medians of the quaternion-valued PPGs are computed. Third, the PCA is employed for denoising the quaternion-valued medians. Finally, the denoised quaternion-valued medians are employed as the features and the random forest model is employed for performing non-invasive blood glucose estimation.

### 3.1. PCA-Based Denoising on the Quaternion-Valued Medians

Since the four-channel PPGs are contaminated by noise, the quaternion-valued medians are also contaminated by noise. On the other hand, the SNRs of the principal components with large variances are higher than those with small variances; the SNRs of the four-channel PPGs can be improved via performing PCA. However, in order to reduce the required computational power, PCA is performed on the quaternion-valued medians instead of on the four-channel PPGs. The detailed procedures are as follows:

Step 1: The covariance matrix of the four-channel PPGs is computed. Then, the eigen vectors of this covariance matrix are found.
Step 2: The four-channel PPGs are treated as the quaternion-valued PPGs. Then, the quaternion-valued medians of the quaternion-valued PPGs are computed as described in Section 2.2.2.
Step 3: The existing quaternion-valued medians are converted to the four-dimensional real-valued vectors. Then, these four-dimensional real-valued vectors are projected to some of the eigen vectors found in Step 1. Let k be the total number of the eigen vectors to be projected. Let $U_{A}=\left[\begin{array}{ccc} u_{1} & \cdots & u_{k} \end{array}\right]$ be the transformation matrix for performing the dimension reduction and the noise reduction. To determine the value of *k*, this paper empirically evaluates the regression errors yielded by the various values of *k*. In particular, *k* is set to 1, 2, and 3. Then, the computer numerical simulations are performed and the optimal value of *k* corresponding to the lowest regression error is found. This approach ensures that the chosen total number of principal components can maximize the effectiveness of the model while the required computational power is kept to the minimum. Here, it is found that the optimal value of *k* is 1.
Step 4: The low-dimensional real-valued vectors are projected back to the four-dimensional real-valued vectors.
Step 5: The reconstructed four-dimensional real-valued vectors are mapped to the quaternion-valued medians and they are employed as the features for performing the blood glucose estimation.

It is worth noting that the quaternion-valued algebra exploits the correlations among the various channels of the quaternion-valued PPGs [12]. Hence, it can achieve a higher regression accuracy compared to that based on the individual channels of the PPGs.

### 3.2. Regression Model

The random forest consists of a set of decision trees [20]. Here, bootstrap sampling is employed for selecting the feature vectors and the features are randomly selected. Each decision tree makes an individual decision. By integrating the decisions made by the individual trees, the random forest can reduce the effects of imbalanced data and prevent the occurrence of overfitting. As a result, it can enhance both the robustness and the accuracy of both the classification and the regression tasks. Hence, it is particularly useful for handling complicated machine learning problems. Because of these advantages, it is widely studied by the machine learning community and applied to many practical applications.

In this paper, a random forest is employed for performing non-invasive blood glucose estimation. In particular, it contains 100 decision trees because 100 decision trees can usually achieve a good balance between the model accuracy and the computational power. Here, the final decision of the random forest is taken as the majority vote of the individual decisions. Moreover, this paper sets the minimum number of the samples in the leaf node to 1. The above parameters are chosen because they are the common settings used in many practical applications [21].

## 4. Computer Numerical Simulation Results

### 4.1. Datasets

**Figure 2:**
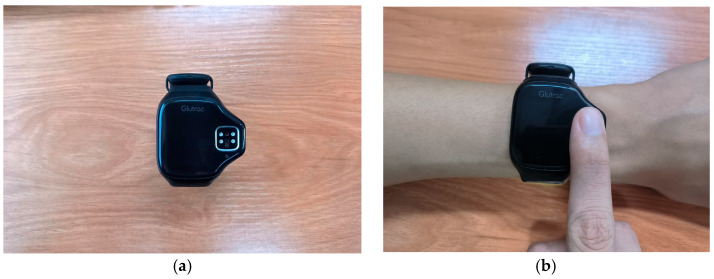
The smart watch used for acquiring the four-channel PPGs. (**a**) The external appearance of the smart watch. (**b**) Illustration of how the device is worn on the wrist during the signal acquisition process.

In this paper, a four-channel smart watch is used as a device for acquiring the four-channel PPGs. Figure 2a shows the external appearance of the smart watch. Here, two light-emitting diodes (LEDs) emit lights with wavelengths equal to 1450 nm, while the other two LEDs emit lights with wavelengths equal to 1650 nm. Each channel of the PPGs is sampled at 50 Hz. The smart watch allows the transmission of the acquired PPGs to the mobile handset. The use of four channels enables the capture of signals at the same two wavelengths (1450 nm and 1650 nm) from different sensor positions on the fingertip. This configuration increases the diversity of the data, helping to improve signal quality by reducing noise and motion artifacts. By acquiring multiple signals from the same wavelengths but different sensor placements, we enhance the robustness and accuracy of the blood glucose estimation, offering complementary information that contributes to the effectiveness of the proposed method.

In the experiment, the fingertip is placed on the surface of the NIR sensors for acquiring the PPGs. Figure 2b shows how the device is worn on the wrist during the signal acquisition process. Here, the acquisition period lasts for 60 s. Hence, the length of each channel’s PPG is 3000 samples. The BGLs are systematically monitored at four fixed time instants at each day. In particular, the first measurement is taken at 8:30 am with the subjects in a fasted state. The second measurement is conducted at 1:00 pm, which is one hour after lunch. The third measurement is taken at 4:30 pm which is between the lunch time and the dinner time. Here, nothing has been eaten during the tea time. Finally, the fourth measurement is taken at 8:00 pm, which is one hour after dinner. In order to obtain a wider range of BGLs closer to the values acquired from the various types of subjects, including hypoglycemia subjects, healthy subjects, and hyperglycemia subjects, the diets of the subjects are artificially altered by including different amounts of carbohydrates in the diets. More precisely, the data acquisition process lasted for 12 days structured into three distinct phases with each phase lasting for 4 days and the amounts of the carbohydrates in the diets in different phases being different. Here, the BGLs acquired in the initial phase are closer to the values acquired from the hypoglycemia subjects. To achieve this goal, the subjects consume a ketogenic diet. The BGLs acquired in the second phase are closer to the values acquired from the healthy subjects. To achieve this goal, the subjects consume a standard diet. The BGLs acquired in the third phase are closer to the values acquired from the hyperglycemia subjects. To achieve this goal, the subjects consume a drink with 300 mL of cola after consuming a standard diet.

In order to evaluate the effectiveness and the robustness of our proposed method, two sets of PPGs are acquired. Here, these two different sets of PPGs are acquired from two different groups of subjects at two different seasons in a year. In particular, the first dataset includes 270 measurements. More precisely, the PPGs and the invasive BGLs were acquired from 18 subjects in May 2022. The ratio of the total number of male subjects to female subjects was three. The age range of the subjects was from 18 years old to 49 years old. The body mass index (BMI) of the subjects ranged from 19.67 to 26.33. On the other hand, the second dataset includes 490 measurements. More precisely, the PPGs and the invasive BGLs were acquired from eight subjects in December 2022. The ratio of the total number of male subjects to female subjects was 2.6. The age range of the subjects was from 20 to 49 years old. The range of the BMI of the subjects was from 17.85 to 27.76. In addition, the ratio of the total number of training feature vectors to test feature vectors was 70%.

### 4.2. Performance Metrics

This paper employs three different performance metrics for evaluating the effectiveness of the various non-invasive blood glucose estimation algorithms. They are the mean absolute error (MAE), the root mean squares error (RMSE), and the MARD. These performance metrics evaluate the discrepancies between the actual BGLs and the corresponding estimated values. More precisely, let ${\hat{y}}_{i}$ be the estimated BGL, $y_{i}$ be the actual BGL, and n be the total number of the test data. The formulas for these performance metrics are

(11) $$MAE=\frac{1}{n}\sum_{i=1}^{n}\left|{\hat{y}}_{i}-y_{i}\right|$$

(12) $$RMSE=\sqrt{\frac{1}{n}\sum_{i=1}^{n}{\left(\hat{y_{i}}-y_{i}\right)}^{2}}$$

and

(13) $$MARD=\frac{1}{n}\sum_{i=1}^{n}\frac{\left|\hat{y_{i}}-y_{i}\right|}{y_{i}}$$

Obviously, lower values refer to more accurate non-invasive blood glucose estimations. Among these three performance metrics, the MARD is the most common one used for evaluating the performance of non-invasive blood glucose estimation algorithms.

Furthermore, the Clarke error grid [22] is also employed for evaluating the effectiveness of the various non-invasive blood glucose estimation algorithms. The estimated BGLs are plotted against the true BGLs. Here, the grid is divided into five different regions, namely, region A to region E [23]. Region A is the region where the estimated BGLs are within an acceptable range from the true BGLs. Since the measurements have high accuracies, the clinical decisions are unaltered and there is no clinical risk imposed to the patients. Region B is the region where the estimated BGLs are within minor deviations from the acceptable range of the true BGLs. Although the estimation errors are obvious, there is still no risk to clinical decisions. Region C is the region where the estimated BGLs are within great deviations from the acceptable range of the true BGLs. Since the estimation errors are large, there is the potential for these to lead to inappropriate clinical decisions. However, the impacts on patient safety are limited. Region D is the region where the estimated BGLs are markedly erroneous compared to the true BGLs. In this case, the clinical errors are substantial and there are considerable risks imposed on patient health. Hence, this non-invasive blood glucose estimation algorithm is unreliable for clinical use. Region E is the region where the estimated BGLs are severely inaccurate compared to the true BGLs. In this case, there is the potential to be life threatening. Hence, this non-invasive blood glucose estimation algorithm is entirely unacceptable for clinical use. By evaluating the performances of the various non-invasive blood glucose estimation algorithms using the Clarke error grid, the researchers and the clinicians can accurately assess the risks of employing non-invasive blood glucose estimation algorithms for clinical use. Hence, evaluating the effectiveness of the non-invasive blood glucose estimation algorithms via the Clarke error grid can ensure the safety of the patients.

### 4.3. Performances Yielded by the Random Forest Based on Applying the PCA to the Various Quaternion-Valued Medians

In this session, PCA subspace dimensions 1 through 3 are referred to as PCA-1, PCA-2, and PCA-3, respectively. The four quaternion median formulas introduced in Section 2, specifically Formulas (7)–(10), are represented by X-M, X-K, X-O, and X-G. Each figure comprises 16 box plots, divided into four groups from left to right according to different quaternion medians. Within each group, the box plots represent the following cases, in order: (i) without PCA processing, (ii) projection into a one-dimensional PCA subspace, (iii) projection into a two-dimensional PCA subspace, and (iv) projection into a three-dimensional PCA subspace.

**Figure 3:**
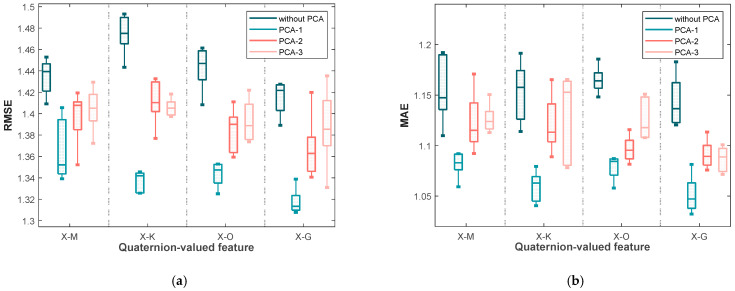
The box plots of (**a**) the RMSE values and (**b**) the MAE values yielded by the various blood glucose estimation methods based on four quaternion-valued medians and these four quaternion-valued medians projected to different subspaces with different dimensions by the PCA for the measurements in the first dataset.

Figure 3 illustrates the RMSE and MAE values for blood glucose estimation based on these four quaternion-valued medians and their projections into different PCA subspaces using the first dataset. It can be observed that, for each quaternion median, projecting into a one-dimensional subspace (PCA-1) yields the lowest RMSE and MAE median values, along with relatively narrow interquartile ranges, indicating both accuracy and stability. In contrast, the worst performance—both in terms of median error and variability—is observed without PCA processing (i.e., using the original four-dimensional quaternion medians). This supports the idea that applying PCA effectively removes redundant information among the components of the quaternion representations, enhancing the predictive performance. Notably, among all medians, X-G yields the lowest RMSE and MAE values under PCA-1, suggesting its superior effectiveness as a feature extraction strategy.

In addition to the median performance, the variability of the results is also informative. Specifically, the differences between the upper and lower quartiles of RMSE and MAE are significantly smaller under PCA-1 for most quaternion medians (X-M, X-K, and X-O), indicating consistent performance across trials. However, for X-G under PCA-1, while the median is the lowest, the interquartile range is slightly wider, implying that although it provides the best average performance, its variability may be slightly higher than the others.

**Figure 4:**
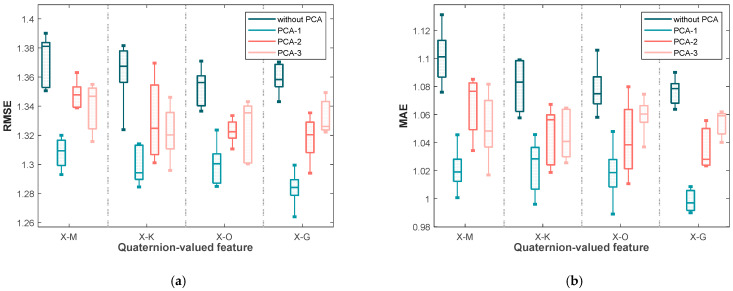
The box plots of (**a**) the RMSE values and (**b**) the MAE values yielded by the various blood glucose estimation methods based on four quaternion-valued medians and these four quaternion-valued medians projected to different subspaces with different dimensions by the PCA for the measurements in the second dataset.

Similarly, Figure 4 displays the RMSE and MAE values for the second dataset. The trends are consistent with those observed in the first dataset: the lowest errors and narrowest interquartile ranges are generally obtained under PCA-1, particularly for X-G. This consistency across datasets confirms that applying PCA to quaternion-valued medians not only improves accuracy but also enhances robustness in non-invasive blood glucose estimation.

### 4.4. Comparison to the State of the Art Methods

For evaluating the effectiveness of applying the PCA to the quaternion-valued medians, the state of the art methods are compared. Here, the quaternion-valued medians are first represented as the four-dimensional real-valued feature vectors. Second, these four-dimensional real-valued feature vectors are projected to the one-dimensional real-valued numbers. Third, these one-dimensional real-valued numbers are projected back to the four-dimensional real-valued feature vectors. Here, as there are four quaternion-valued medians, the dimension of the overall feature vectors is 16. Finally, these 16-dimensional overall feature vectors are employed for performing non-invasive blood glucose estimation via the random forest.

These 16-dimensional overall feature vectors are compared to the feature vectors employed in the state of the art methods for performing non-invasive blood glucose estimation. In particular, the time-domain statistical features are extracted from three PPGs acquired using three different LEDs with three different wavelengths [4]. Moreover, the aforementioned features including the logarithmic energy entropy, the KTE, the spectral entropy, and the AR coefficients are extracted from the PPGs [5]. Furthermore, a variety of the time-domain and the frequency-domain features such as the zero crossing rate, the auto-correlation coefficients, the power spectral density coefficients, the KTE, the spectral coefficients, the wavelet coefficients and the AR coefficients are extracted from the PPGs [7]. In addition, the MFCCs are extracted from the PPGs [8]. In addition, the time-domain HRV features and the frequency-domain HRV features are extracted from the PPGs [9]. Table 1 and Table 2 show the results yielded by these methods using SVR based on the PPGs in the first dataset and the second dataset, respectively. Similarly, Table 3 and Table 4 present the corresponding results obtained using the RF model. Across both datasets, the proposed method consistently achieves the lowest MARD, RMSE, and MAE values compared to existing approaches, regardless of the regression model employed. Furthermore, the results obtained under the RF model exhibit lower error metrics in the majority of experimental settings relative to those under SVR, suggesting that RF may offer enhanced predictive capability for non-invasive blood glucose estimation within the context of this study. These findings collectively demonstrate the effectiveness and robustness of the proposed method, as well as the relative advantage of adopting RF over SVR in this application.

**Figure 5:**
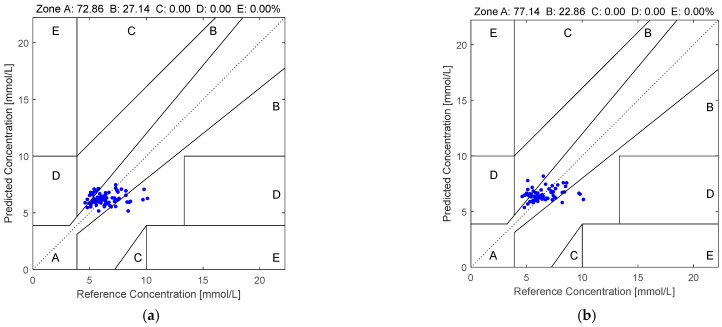
The Clarke error grids yielded by the quaternion-valued medians projected to the low-dimensional space via the PCA according to the PPGs in the first dataset: (**a**) SVR and (**b**) RF.

**Figure 6:**
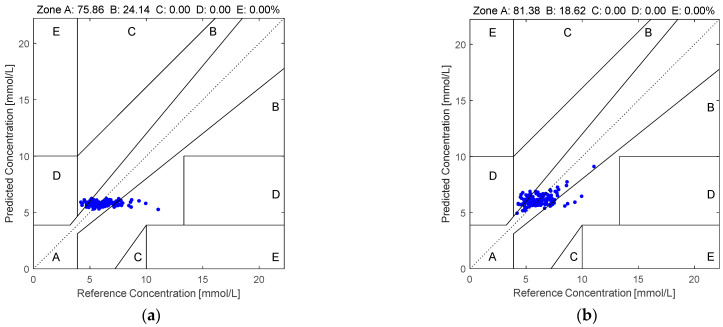
The Clarke error grids yielded by the quaternion-valued medians projected to the low-dimensional space via the PCA according to the PPGs in the second dataset: (**a**) SVR and (**b**) RF.

Figure 5 illustrates the Clarke error grid analysis of the proposed method applied to the first dataset using two different regression models: SVR in subfigure (a) and RF in subfigure (b). Similarly, Figure 6 presents the corresponding results on the second dataset. As observed from the Clarke error grids, the predicted points generated by the RF-based model are more densely distributed along the diagonal line compared to those generated by the SVR-based model, indicating a stronger correlation between the estimated and reference blood glucose values. Moreover, a higher proportion of points fall within region A when using the RF model, further demonstrating the effectiveness and robustness of the proposed method.

### 4.5. Discussion

In this study, we proposed a quaternion-valued framework for non-invasive blood glucose estimation based on multi-channel PPG signals. Our method integrates four-channel PPG inputs into a quaternion-valued signal structure, enabling the joint representation of inter-channel information. The results from two datasets demonstrate that the proposed method significantly outperforms conventional multi-channel approaches, achieving lower MARD, RMSE, and MAE values, along with higher proportions of estimations falling within region A of the Clarke error grid.

The observed performance improvements can be attributed to two key factors. First, the quaternion representation effectively preserves the spatial and temporal relationships among multiple PPG channels, which are often ignored when channels are processed independently. This is particularly important given the inherent correlations among synchronously acquired multi-channel signals. By modeling these inter-channel interactions within a compact four-dimensional framework, our method captures richer and more discriminative features that contribute to improved regression accuracy. Second, the application of PCA on quaternion-derived features not only reduces dimensionality and suppresses noise but also helps highlight the most informative signal components, thereby enhancing robustness and generalization across datasets.

Despite the promising results, this study has several limitations. First, the requirement for four-channel PPG acquisition may limit the applicability of the method to simpler or lower-cost hardware systems, especially in wearable or portable settings. Second, although the datasets used in our experiments are diverse and include multiple subjects, they may not fully represent the variability found in broader populations. Additionally, our evaluation was conducted under relatively controlled conditions, and the impact of real-world noise sources such as motion artifacts, skin tone variability, or sensor misalignment was not extensively tested. These factors may influence model performance in practical deployments.

Looking ahead, the proposed quaternion-based framework has the potential to be extended to other non-invasive health monitoring applications. In particular, physiological markers such as blood pressure and blood lipid levels are also influenced by multi-channel signals, including multi-wavelength PPG or multimodal biosensing data. Given that inter-channel coupling is also relevant in these domains, the quaternion representation could offer similar benefits. Future work will focus on evaluating the adaptability of this method to such tasks, validating performance under real-world conditions, and exploring its integration into embedded systems for continuous and real-time health monitoring.

## 5. Conclusions

This paper employs quaternion-valued medians as features for performing non-invasive blood glucose estimation. In addition, PCA is employed for suppressing the noise in the quaternion-valued medians. First, the four-channel PPGs are acquired and they are used to form the quaternion-valued PPGs. Second, the existing quaternion-valued medians are computed and they are mapped to the four-dimensional real-valued vectors. Third, PCA is used to project these four-dimensional real-valued vectors to the low-dimensional real-valued vectors. Then, the low-dimensional real-valued feature vectors are mapped back to the four-dimensional real-valued feature vectors. Finally, the random forest is used for performing the blood glucose estimation. Two datasets are employed for evaluating the effectiveness of our proposed method. The computer numerical simulation results show that our proposed method yields an MARD value, RMSE value, MAE value, and percentage of the pairs of the estimated BGLs and the real BGLs falling in region A of the Clarke error grid at 0.1498, 1.2175, 0.9586, and 77.14%, respectively, for the PPGs in the first dataset as well as 0.1369, 1.1445, 0.8572, and 81.38%, respectively, for the PPGs in the second dataset. Compared to the existing methods, our proposed method is more effective and robust.

## Figures and Tables

**Table 1:** The comparisons of our proposed method to the existing methods using SVR based on the PPGs in the first dataset.

Features	MARD	RMSE	MAE	A(%)	B(%)
Time-domain statistical features [4]	0.1748	1.4408	1.1200	58.57	41.43
Logarithmic energy entropy, KTE, spectral entropy, AR coefficients [5]	0.1630	1.3789	1.0919	60	40
Time-domain features, frequency-domain features, KTE [7]	0.1679	1.5175	1.1374	67.14	32.86
MFCCs [8]	0.1638	1.3330	1.0408	64.29	35.71
Time-domain HRV features, frequency-domain HRV features [9]	0.1678	1.4509	1.1055	64.29	35.71
Quaternion-valued medians projected to the low-dimensional space via the PCA (our proposed method)	**0.1535**	**1.3274**	**1.0143**	**72.86**	**27.14**

**Table 2:** The comparisons of our proposed method to the existing methods using SVR based on the PPGs in the second dataset.

Features	MARD	RMSE	MAE	A(%)	B(%)
Time-domain statistical features [4]	0.1526	1.2185	0.9659	69.66	30.34
Logarithmic energy entropy, KTE, spectral entropy, AR coefficients [5]	0.1502	1.1367	0.9115	70.34	29.66
Time-domain features, frequency-domain features, KTE [7]	0.1465	1.2118	0.9117	70.34	29.66
MFCCs [8]	0.1497	1.1912	0.9145	71.72	28.28
Time-domain HRV features, frequency-domain HRV features [9]	0.1458	1.2407	0.9290	74.48	25.52
Quaternion-valued medians projected to the low-dimensional space via the PCA (our proposed method)	**0.1439**	**1.2097**	**0.9038**	**75.86**	**24.14**

**Table 3:** The comparisons of our proposed method to the existing methods using RF based on the PPGs in the first dataset.

Features	MARD	RMSE	MAE	A(%)	B(%)
Time-domain statistical features [4]	0.1663	1.3387	1.0457	71.43	28.57
Logarithmic energy entropy, KTE, spectral entropy, AR coefficients [5]	0.1607	1.2808	1.0253	72.86	27.14
Time-domain features, frequency-domain features, KTE [7]	0.1610	1.2889	1.0053	71.43	28.57
MFCCs [8]	0.1647	1.2944	1.0489	68.57	31.43
Time-domain HRV features, frequency-domain HRV features [9]	0.1612	1.2868	1.0400	70	30
Quaternion-valued medians projected to the low-dimensional space via the PCA (our proposed method)	**0.1498**	**1.2175**	**0.9586**	**77.14**	**22.86**

**Table 4:** The comparisons of our proposed method to the existing methods using RF based on the PPGs in the second dataset.

Features	MARD	RMSE	MAE	A(%)	B(%)
Time-domain statistical features [4]	0.1498	1.1901	0.9243	71.72	28.28
Logarithmic energy entropy, KTE, spectral entropy, AR coefficients [5]	0.1469	1.1754	0.8971	75.86	24.14
Time-domain features, frequency-domain features, KTE [7]	0.1479	1.1563	0.9095	72.41	27.59
MFCCs [8]	0.1464	1.1575	0.9002	73.10	26.90
Time-domain HRV features, frequency-domain HRV features [9]	0.1455	1.1527	0.8964	76.55	23.45
Quaternion-valued medians projected to the low-dimensional space via the PCA (our proposed method)	**0.1369**	**1.1445**	**0.8572**	**81.38**	**18.62**

## Data Availability

The data that support the findings of this study are available from the corresponding author upon reasonable request.
